# Neurostructural correlates of hope: dispositional hope mediates the impact of the SMA gray matter volume on subjective well-being in late adolescence

**DOI:** 10.1093/scan/nsaa046

**Published:** 2020-04-30

**Authors:** Song Wang, Yajun Zhao, Jingguang Li, Han Lai, Chen Qiu, Nanfang Pan, Qiyong Gong

**Affiliations:** 1 Huaxi MR Research Center, Department of Radiology, West China Hospital of Sichuan University, Chengdu 610041, China; 2 Psychoradiology Research Unit of Chinese Academy of Medical Sciences (2018RU011), the Functional & Molecular Imaging Key Laboratory of Sichuan Province, West China Hospital of Sichuan University, Chengdu 610041, China; 3 Department of Psychoradiology, Chengdu Mental Health Center, Chengdu 610036, China; 4 School of Sociology and Psychology, Southwest Minzu University, Chengdu 610041, China; 5 College of Teacher Education, Dali University, Dali 671003, China; 6 Department of Psychology, The Faculty of Social Science, The University of Hong Kong, Pokfulam 999077, Hong Kong

**Keywords:** dispositional hope, subjective well-being, structural magnetic resonance imaging, supplementary motor area, adolescent, psychoradiology, mental health

## Abstract

There has been increasing interest in identifying factors to predict subjective well-being in the emerging field of positive psychology over the past two decades. Dispositional hope, which reflects one’s goal-directed tendencies, including both pathway thinking (planning to meet goals) and agency thinking (goal-directed determination), has emerged as a stable predictor for subjective well-being. However, the neurobiological substrates of dispositional hope and the brain-hope mechanism for predicting subjective well-being remain unclear. Here, we examined these issues in 231 high school graduates within the same grade by estimating cortical gray matter volume (GMV) utilizing a voxel-based morphometry method based on structural magnetic resonance imaging. Whole-brain regression analyses and prediction analyses showed that higher dispositional hope was stably associated with greater GMV in the left supplementary motor area (SMA). Furthermore, mediation analyses revealed that dispositional hope mediated the relation between left SMA volume and subjective well-being. Critically, our results were obtained after adjusting for age, sex, family socioeconomic status and total GMV. Altogether, our study presents novel evidence for the neuroanatomical basis of dispositional hope and suggests an underlying indirect effect of dispositional hope on the link between brain gray matter structure and subjective well-being.

## Introduction

With the enormous growth of positive psychology in the past two decades (Lopez *et al.,* 2018), there is an increasing interest in identifying predictors for subjective well-being, which reflects an individual’s evaluation of her/his life, including both affective (i.e. hedonic balance) and cognitive aspects (i.e. satisfaction with life) (Diener, 2000; Diener and Ryan, 2009; Diener *et al.,* 2018). It is well established that a bulk of biopsychosocial factors, such as genetic predispositions (Okbay *et al.,* 2016), cultural circumstances (Steel *et al.,* 2018), economic status (Dolan *et al.,* 2008) and personality traits (DeNeve and Cooper, 1998) are reliable predictors of subjective well-being. As one of the key personality factors, dispositional hope is one’s tendency to apply a cognitive-focused process, including both pathway thinking (planning to meet goals) and agency thinking (goal-directed determination) (Snyder *et al.,* 1991; Snyder, 2002). Findings from numerous studies have suggested that dispositional hope is stably linked to subjective well-being among different populations. For instance, many cross-sectional studies have demonstrated a positive relation between dispositional hope and subjective well-being among adults (Park *et al.,* 2004; Gallagher and Lopez, 2009; Demirli *et al.,* 2015; Yalcin and Malkoc, 2015; Satici, 2016) and adolescents and children (Gilman *et al.,* 2006; Bronk *et al.,* 2009; Wong and Lim, 2009; Merkas and Brajsa-Zganec, 2011; Blanca *et al.,* 2018). Furthermore, several longitudinal studies have revealed that dispositional hope plays a predictive role in subjective well-being (Ciarrochi *et al.,* 2007; Marques *et al.,* 2013; Ng *et al.,* 2014; Ciarrochi *et al.,* 2015; Heinitz *et al.,* 2018). Additionally, evidence from an intervention study showed that a hope-based training program can improve individuals’ levels of satisfaction with life, suggesting that dispositional hope may be a potential psychological factor that can be shaped to enhance subjective well-being (Proyer *et al.,* 2013). In summary, dispositional hope is a potential variable for predicting subjective well-being. Here, using structural magnetic resonance imaging (sMRI), we assessed the neuroanatomical correlates of dispositional hope and then examined the underlying brain-hope mechanism in predicting subjective well-being.

Although prior studies have confirmed the predictive role of dispositional hope in subjective well-being, less work has investigated the neural mechanisms underlying dispositional hope. Theoretically, the concept of dispositional hope mainly includes two aspects: pathway thinking enables people to solve problems and meet goals by producing possible or different solutions; agency thinking empowers people to pursue specific goals by initiating and sustaining motivations (Snyder *et al.,* 1991; Snyder, 2002). Therefore, the neurobiological basis underlying dispositional hope has been hypothesized to be linked with the functioning of the prefrontal cortex (PFC) (Wang, Xu *et al.,* 2017), which has been shown to be crucial for goal-directed behavior and thinking, the initiation and maintenance of motivation, and problem solving-related processes (Waltz *et al.,* 1999; Miller and Cohen, 2001; Hasselmo, 2005; Kouneiher *et al.,* 2009). Nevertheless, to our knowledge, only one empirical study has directly explored the neurofunctional substrates of dispositional hope by using resting-state functional MRI (rsfMRI); this study observed that dispositional hope was negatively related to coordinated resting-state brain activity in the medial orbitofrontal cortex (OFC) (Wang, Xu *et al.,* 2017). Unlike the rsfMRI technique, which reflects the functional architecture of the brain measured with the spontaneous low-frequency fluctuation in the blood oxygenation level-dependent signal (Biswal, 2012; Lee *et al.,* 2013), the sMRI technique measures the structural characteristics of the brain and has been widely used to investigate the neuroanatomical correlates of human mind and behavior (DeYoung and Gray, 2009; Kanai and Rees, 2011; Lerch *et al.,* 2017). Considering that no studies have examined the association between dispositional hope and brain structure, the first goal of this study was to identify the brain regions whose gray matter structure was associated with dispositional hope in a large group of adolescents (N > 200) within a narrow age range, which may offer sufficient statistical power for whole-brain analyses (Mar *et al.,* 2013; Mackey *et al.,* 2015).

Compared with the few neuroimaging studies on dispositional hope, a relatively large number of studies have examined the neural basis underlying subjective well-being. Evidence from the existing literature has suggested that subjective well-being is also mainly associated with the function and structure of the PFC (King, 2019). For example, electroencephalography research revealed that alpha power in the frontocentral region was linked to subjective well-being (Urry *et al.,* 2004). Using a perfusion MRI technique, Hermes *et al.* (2011) observed a negative association of positive affect with baseline cerebral blood flow in the anterior cingulate cortex (ACC). Similarly, another study based on positron emission tomography found that the baseline glucose metabolism in the medial and lateral PFC was associated with positive emotionality, which is a psychological construct closely linked to hedonic balance (Volkow *et al.,* 2011). Moreover, abundant rsfMRI studies has revealed widespread associations between subjective well-being and coordinated spontaneous brain activity in several PFC regions, including the ACC, OFC and superior frontal gyrus (SFG) (Kong, Hu *et al.,* 2015; Kong, Wang *et al.,* 2015; Kong *et al.,* 2016; Kong *et al.,* 2018). Furthermore, it has been reported that subjective well-being is also related to brain gray matter structures in the medial and lateral PFC regions (Kong, Ding *et al.,* 2015; Matsunaga *et al.,* 2016; Zhu *et al.,* 2018). Given these findings and the crucial role of dispositional hope in subjective well-being, the second goal of this study was to explore whether the brain areas related to dispositional hope could be linked to subjective well-being and then to examine the mediating relationship among brain structure, dispositional hope and subjective well-being.

To achieve these goals, we conducted sMRI scans on participants and evaluated their levels of dispositional hope and subjective well-being with standard tests. Here, we used a voxel-based morphometry (VBM) approach to estimate brain gray matter volume (GMV) (Ashburner and Friston, 2000; Ashburner and Friston, 2001). Particularly, we chose GMV as our brain structural metric as this index is a comprehensive measure based on cortical thickness and cortical surface area that may reflect the numbers and sizes of unmyelinated neurons and glial cells, along with the volume of the synapses (Takeuchi *et al.,* 2012, 2014), and this index has also been widely used to identify structural features of the brain that underlie a personality construct (DeYoung and Gray, 2009; Lai *et al.,* 2019). First, whole-brain regression analyses were conducted to identify the brain areas related to dispositional hope. Given the prior neurobiological hypothesis and findings regarding dispositional hope (Wang, Xu *et al.,* 2017), we speculated that GMV in some PFC regions may be linked to dispositional hope. Second, correlation analyses and mediation analyses were performed to probe the associations between dispositional hope, subjective well-being and GMV. Given the predictive role of dispositional hope in subjective well-being (Ciarrochi *et al.,* 2007; Marques *et al.,* 2013; Ng *et al.,* 2014; Ciarrochi *et al.,* 2015; Heinitz *et al.,* 2018) and the previous neuroimaging findings on subjective well-being (King, 2019), we further conjectured that some brain regions linked with dispositional hope may be related to subjective well-being and that there may be an indirect effect of dispositional hope on the association between GMV and subjective well-being.

## Methods

### Participants

The current data were collected as part of a larger project to explore the behavioral and neurobiological mechanisms underlying personality traits, mental health and academic achievement among Chinese adolescents (Wang, Kong *et al.,* 2017; Li, Zhao *et al.*, 2018; Wang, Dai *et al.,* 2018; Wang *et al.,* 2019). The participants comprised 231 students (121 females, age range = 16–20 years) who had recently completed the 12th grade at several local public high schools in Chengdu, China. Each participant first completed a battery of behavioral tests with a paper and pencil form and then underwent sMRI scans. All participants were native speakers of Mandarin Chinese and right-handed as assessed with the Edinburgh Handedness Inventory (Oldfield, 1971) and had no previously diagnosed neuropsychiatric, developmental or sleep disorders (Wang *et al.,* 2019). All participants and their guardians provided written informed consent prior to testing in accordance with the Declaration of Helsinki. The study protocol was approved by the local research ethics committee of West China Hospital of Sichuan University. Notably, we have performed several other analyses on these participants (e.g. analyses regarding the association between GMV and delay discounting (Wang, Kong *et al.,* 2017), trait grit (Wang, Dai *et al.,* 2018) and academic performance (Wang, Zhou *et al.,* 2017); analyses on the link between resting-state brain activity and perceived stress (Wang *et al.,* 2019), dispositional optimism (Wang, Zhao *et al.,* 2018) and dispositional hope (Wang, Xu *et al.,* 2017)) and the corresponding results have been reported in these studies.

### Behavioral tests

#### Dispositional hope

The participants’ levels of dispositional hope were measured using the Chinese version of the Dispositional Hope Scale (DHS) (Sun *et al.,* 2012; Li, Mao *et al.,* 2018). The scale has two dimensions: pathway thinking and agency thinking, each with four items (Snyder *et al.,* 1991). The participants rated items on a four-point response scale (1 = definitely false, 4 = definitely true). The score for the DHS was derived from summing the two dimension scores based on the ratings of the corresponding items, with higher scores suggesting higher dispositional hope. Evidence from previous studies has suggested that the DHS total score is suitable for use in empirical investigations (Snyder *et al.,* 1991; Chang *et al.,* 2016; Wang, Xu *et al.,* 2017). The Chinese version of the DHS has shown satisfactory psychometric properties in different populations (Sun *et al.,* 2012; Li, Mao *et al.,* 2018; Chang *et al.,* 2019). Here, the internal consistency for the DHS was adequate (*α* = 0.75).

#### Hedonic balance

To evaluate the levels of hedonic balance, we employed the Positive and Negative Affect Schedule (PANAS), which is a popular measure for assessing general affective characteristics (Watson *et al.,* 1988). The PANAS contains 10 positive affect words (e.g. ‘active’) and 10 negative affect words (e.g. ‘hostile’). The individuals were asked to rate the extent to which they generally feel that particular affect on a five-point response format (1 = not at all, 5 = extremely). The psychometric properties of the Chinese version of the PANAS have been well established in Chinese populations (Chen, 2004; Kong, Ding *et al.,* 2015; Wang, Xu *et al.,* 2017). Here, the internal consistency for the PANAS was satisfactory (for positive affect, *α* = 0.86; for negative affect, *α* = 0.84). According to standard conventions (Diener *et al.,* 1995; Schimmack *et al.,* 2002; Kong *et al.,* 2018), the hedonic balance can be reflected by the relative amount of positive affect to negative affect. Thus, we calculated the hedonic balance scores by subtracting the negative affect scores from the positive affect scores, with higher scores suggesting a higher likelihood of experiencing positive affect.

**Satisfaction with life.** To assess the levels of satisfaction with life, the Satisfaction with Life Scale (SWLS) (Diener *et al.,* 1985) was administered to all participants. The SWLS is a unidimensional scale and includes five items that are rated on a seven-point scale (1 = strongly disagree, 7 = strongly agree). The SWLS score was obtained by summing the ratings of all items, with higher scores suggesting higher satisfaction with life. This scale has been repeatedly used in different Chinese populations and has been shown to have good validity and reliability (Bai *et al.,* 2011; Kong *et al.,* 2012; Kong, Ding *et al.,* 2015). Here, the internal consistency for the SWLS was adequate (*α* = 0.75).

#### Family socioeconomic status (SES)

Considering that family SES is found to be stably related to dispositional hope (Snyder, 2002; Dixson *et al.,* 2018), brain structure (Hackman and Farah, 2009; Farah, 2017) and subjective well-being (Haring *et al.,* 1984; Quon and McGrath, 2014), we employed the Socioeconomic Status Scale (SSS) (Adler *et al.,* 2000) to exclude the potential effects of family SES on the relations among dispositional hope, GMV and subjective well-being. The scale is a single-item measure presenting participants with a drawing of a 10-rung ladder. Each participant was instructed to indicate the overall level of his/her parents’ education, occupational prestige and income over a range from 1 (bottom rung) to 10 (top rung). Converging evidence has revealed that compared to objective measures, SSS has a better predictive ability for health-linked outcomes (Cundiff and Matthews, 2017). This scale has also been widely used in Chinese populations (Hu *et al.,* 2005; Kong, Wang *et al.,* 2015; Wang, Zhou *et al.,* 2017).

### Image acquisition and preprocessing

#### Image acquisition

We performed the sMRI experiments using a 3.0 T Siemens-Trio Erlangen scanner with a 12-channel head coil. Using a whole-head magnetization-prepared rapid gradient-echo sequence, each participant underwent a T1-weighted structural scan with the following parameters: 176 slices, voxel size = 1 × 1 × 1 mm^3^, matrix size = 256 × 256, slice thickness = 1 mm, flip angle = 9 degrees, inversion time/repetition time/echo time = 900/1900/2.26 ms.

#### Image preprocessing

The pre-processing of images was conducted using Statistical Parametric Mapping software (SPM12) (Friston *et al.,* 2007). All of the images were first displayed in SPM12 to check for gross anatomical abnormalities or artifacts. For a more accurate registration, each image was manually reoriented, aligned to the anterior commissure, and then segmented into three tissue groups (i.e. cerebrospinal fluid, white matter and gray matter) by employing the new segmentation tool in SPM12. Afterward, registration, normalization and modulation analyses were conducted using DARTEL algebra (Ashburner, 2007) in SPM12. The gray matter data were aligned, resampled to 2 × 2 × 2 mm^3^, and then transformed to Montreal Neurological Institute (MNI) space. The inverse Jacobian of the local transformations was used to modulate the segmented gray matter data, which allowed the volume measurements to be preserved. Subsequently, the normalized and modulated data were smoothed with an 8-mm full-width at half-maximum Gaussian kernel. Finally, the resulting images were masked with an absolute threshold masking of 0.2 to remove edge effects around the borders between gray matter and white matter (Kong, Ding *et al.,* 2015; Yao *et al.,* 2018; Wang, Dai *et al.,* 2018). The resulting data representing GMV were adopted in the subsequent analyses.

### Statistical analyses

#### GMV-behavior correlation analysis

To detect the brain regions whose GMV was linked with dispositional hope, we conducted a whole-brain regression analysis using DHS scores as the independent variable (*X*); voxelwise GMV as the dependent variable (*Y*); and sex, age, family SES and total GMV as the control variables. To investigate the association of GMV with two dimensions of dispositional hope (i.e. pathway thinking and agency thinking), we conducted another two whole-brain regression analyses using sex, age, family SES and total GMV as the control variables. To correct for multiple comparisons, we used random field theory (RFT), which considers both peaks and spatial extent by modeling noise as Gaussian random fields (Worsley *et al.,* 1992; Ashburner and Friston, 2000; Mechelli *et al.,* 2005). This approach provided significant clusters of voxels at the familywise error rate of *P <* 0.05 (for a *P*-voxel threshold *<* 0.001). To locate the significant clusters, we employed the slice viewer of REST software, which combines the methods of Harvard–Oxford atlas, Anatomical Automatic Labeling and Brodmann’s area (Song *et al.,* 2011). The detailed procedures for the localization are as follows: http://restfmri.net/forum/sites/default/files/how%20to%20report%20result-2.0.pdf.

#### Prediction analysis

To validate the robustness of the brain-hope association, we implemented a balanced four folds cross-validation procedure through a machine learning method (Supekar *et al.,* 2013; Yang *et al.,* 2016; Wang, Dai *et al.,* 2018; Kong *et al.,* 2019). For the analysis, a linear regression algorithm was performed using DHS scores as the *Y* and GMV of the identified brain region as the *X*. The data were first divided into four folds to guarantee that there were no significant differences among the distributions of these variables across folds. Then, the data from three folds were used to build a linear regression model, with one fold left out. This model was further employed to predict the unused data fold. The value *r*_(predicted, observed)_, which represents the correlation of the actual observed data and the predicted data, was finally obtained after the data for all folds had been predicted. The significance of *r*_(predicted, observed)_ was determined using a non-parametric testing method by generating 5000 surrogate datasets, following the test procedures applied in the previous studies (Supekar *et al.,* 2013; Yang *et al.,* 2016; Wang, Dai *et al.,* 2018; Kong *et al.,* 2019). Sex, age, family SES and total GMV were controlled for in this analysis.

#### Mediation analysis

To evaluate the indirect effect of GMV on subjective well-being through dispositional hope, a mediation analysis was conducted with the PROCESS macro in SPSS (Hayes, 2013). For this analysis, GMV of the identified brain region was considered the *X*, dispositional hope was considered the mediator variable (*M*), and hedonic balance or satisfaction with life was considered the *Y*. The significance of the indirect effect was determined using a bootstrapping method with 5000 iterations. If a 95% confidence interval (CI) did not contain zero, then the indirect effect was significant. Sex, age, family SES and total GMV were controlled for in this analysis.

## Results

### Neurostructural correlates of dispositional hope

Table 1 shows the means, standard deviations (SD) and correlations of all behavioral variables included in this study. Dispositional hope had no significant association with age (*r* = 0.03, *P* = 0.641), sex [*t* (229) = 1.85, *P* = 0.066] or total GMV (*r* = 0.12, *P* = 0.071). A significant correlation was observed between dispositional hope and family SES (*r* = 0.23*, P <* 0.001). We then investigated the neurostructural substrates of dispositional hope.

**Table 1:** Means, SD and correlations of the behavioral variables

Variable	Mean	SD	1	2	3	4	5	6
1. Age	18.48	0.54	1.00					
2. Dispositional hope	21.66	2.69	0.03	1.00				
3. Hedonic balance	10.81	8.43	0.06	0.53^***^	1.00			
4. Positive affect	33.65	5.79	0.01	0.59^***^	0.76^***^	1.00		
5. Negative affect	22.84	5.54	−0.08	−0.18^**^	−0.73^***^	−0.11	1.00	
6. Satisfaction with life	20.90	4.95	−0.09	0.34^***^	0.33^***^	0.25^***^	−0.24^***^	1.00
7. Family SES	5.27	1.49	−0.02	0.23^***^	0.17^**^	0.14^*^	−0.12	0.30^***^

^*^ *P <* 0.05; ^**^P < 0.01; ^***^P < 0.001.

**Fig. 1:**
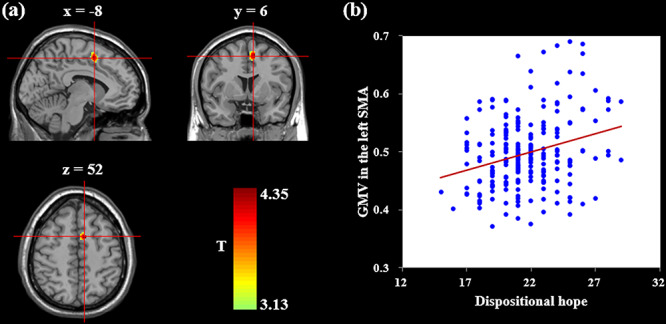
Regional GMV linked to dispositional hope. (a) Brain image showing that dispositional hope is positively correlated with GMV in the left SMA after controlling for sex, age, family SES and total GMV. (b) Scatter plots depicting the correlation between dispositional hope and left SMA volume (*r* = 0.28, *P <* 0.001). The background image was a specific study template in the MNI152 space (i.e. the Ch2 template in the REST slice viewer), to which each participant’s gray matter image was normalized.

The whole-brain regression analysis found that after RFT correction for multiple testing, dispositional hope showed a positive association with GMV in the left supplementary motor area (SMA) extending to SFG (Table 2 and Fig. 1), with sex, age, family SES and total GMV as covariates. The unthresholded statistical maps of this analysis: see Supplemental materials. When all covariates were excluded in this analysis, dispositional hope was still linked to GMV in the left SMA (peak MNI coordinate: [−10, 4, 52], peak *t* = 4.88, *P* < 0.001, cluster size = 1392 mm^3^), after correcting for multiple comparisons with RFT. However, another two whole-brain regression analyses found that no cluster was significantly linked with the two dimensions of dispositional hope (e.g. agency thinking and pathway thinking). For the follow-up analyses, we used the SMA detected from the initial whole-brain regression analysis as a region of interest (ROI) and explored its relation to other variables.

**Table 2:** Brain regions whose GMV correlated with dispositional hope

Region	Side	Peak MNI coordinate			Peak *T* score	Cluster size (mm^3^)
		x	y	z		
SMA	L	−10	4	52	4.35	752

*Note:* L = left. A Gaussian random field approach was employed to determine the regions of significance with the following thresholds: *P* < 0.05 at the cluster level and *P* < 0.001 at the underlying voxel level.

Given that the adolescent brain undergoes continuing maturation with age (Dumontheil, 2016), we next tested whether age can moderate the association between GMV and dispositional hope. At the ROI level, there was no interaction effect of age and dispositional hope on GMV in the left SMA (*△R^2^* = 0.01%, *β* = 0.13, *P* = 0.95), after controlling for sex, age, family SES, total GMV and dispositional hope. At the whole-brain level, we also observed no significant regions for the interaction effect of age by dispositional hope with sex, age, family SES, total GMV and dispositional hope as covariates, after correcting for multiple comparisons with RFT.

We then implemented prediction analyses to check the robustness of the relation between dispositional hope and GMV in the left SMA. After controlling for sex, age, family SES and total GMV, dispositional hope was significantly predicted by GMV in the left SMA [*r*_(predicted, observed)_ = 0.24 *P <* 0.001], indicating that the link between dispositional hope and GMV in the left SMA was stable.

### Dispositional hope linking brain structure and subjective well-being

After evaluating the neurostructural correlates of dispositional hope, we further investigated the potential brain-hope mechanism in predicting subjective well-being measured with the PANAS and SWLS. First, we verified the positive association of dispositional hope with hedonic balance (*r* = 0.50, *P <* 0.001) and satisfaction with life (*r* = 0.31, *P <* 0.001). Second, we tested whether subjective well-being could be linked with GMV in the identified brain regions (i.e. the left SMA). We found a significant association of left SMA GMV with hedonic balance (*r* = 0.18, *P =* 0.008) and satisfaction with life (*r* = 0.14, *P =* 0.032). Sex, age, family SES and total GMV were adjusted for in these analyses.

**Fig. 2:**
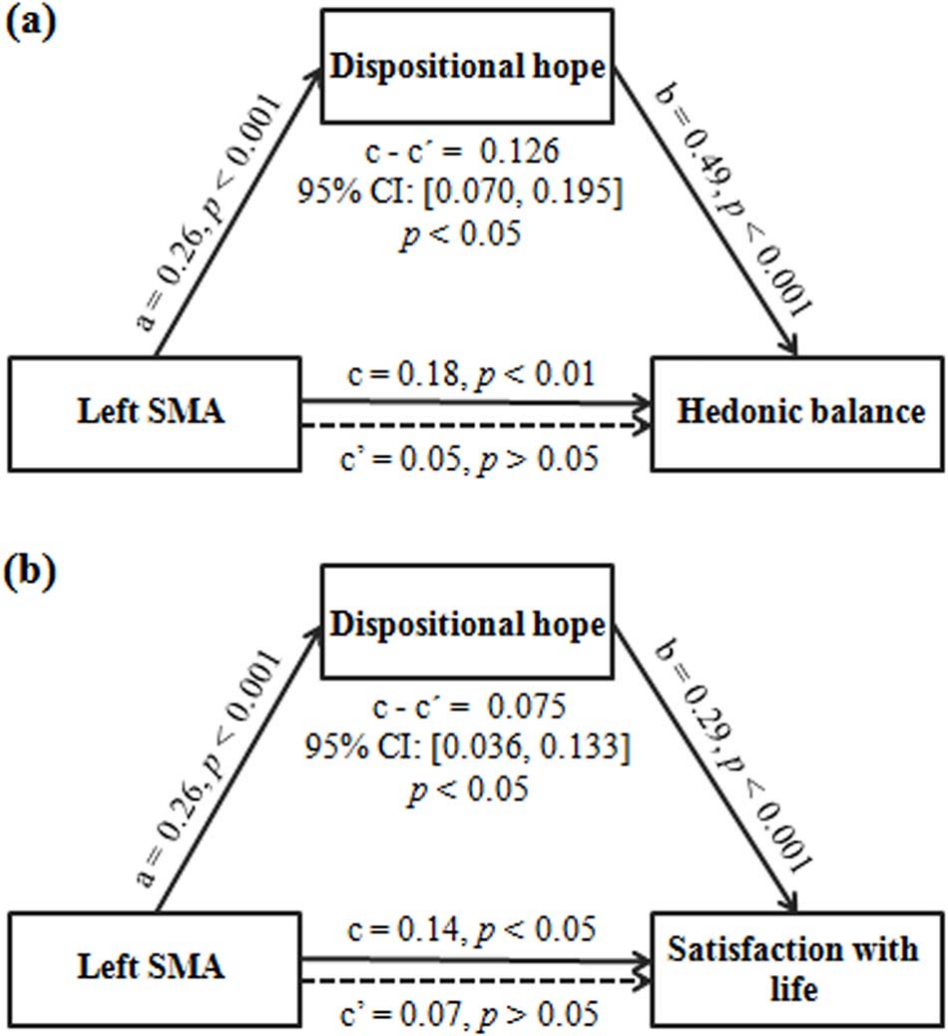
Dispositional hope mediates the effect of left SMA volume on hedonic balance (a) and satisfaction with life (b). Standardized regression coefficients are presented in the path diagrams. Sex, age, family SES and total GMV are controlled for in the models.

We then performed mediation analyses to test whether dispositional hope could mediate the link between GMV and subjective well-being. We found a significant indirect effect of dispositional hope on the association between left SMA GMV and hedonic balance (indirect effect = 0.126, 95% CI = [0.070, 0.195], *P <* 0.05; Fig. 2a) and satisfaction with life (indirect effect = 0.075, 95% CI = [0.036, 0.133], *P <* 0.05; Fig. 2b). Sex, age, family SES and total GMV were adjusted for in these analyses. In summary, dispositional hope may explain covariance between left SMA GMV and subjective well-being.

Furthermore, we conducted several other mediation analyses to explore the directionality of the associations between GMV, dispositional hope and subjective well-being. Particularly, considering that several previous longitudinal studies have indicated that dispositional hope is a stable predictor for subjective well-being (Ciarrochi *et al.,* 2007; Marques *et al.,* 2013; Ng *et al.,* 2014; Ciarrochi *et al.,* 2015; Heinitz *et al.,* 2018), there are three possible mediation models for the association among GMV, dispositional hope and subjective well-being (Model 1: *X* = left SMA, *M* = dispositional hope, *Y* = subjective well-being; Model 2: *X* = dispositional hope, *M* = left SMA, *Y* = subjective well-being; Model 3: *X* = dispositional hope, *M* = subjective well-being, *Y* = left SMA). In addition to Model 1, which was examined in the above analyses, we further examined Models 2 and 3 and found no significant indirect effects in either of these two models. Specifically, left SMA GMV did not mediate the effect of dispositional hope on hedonic balance (indirect effect = 0.013, 95% CI = [−0.016, 0.058], *P >* 0.05) or satisfaction with life (indirect effect = 0.017, 95% CI = [−0.014, 0.057], *P >* 0.05). Similarly, hedonic balance (indirect effect = 0.030, 95% CI = [−0.045, 0.109], *P >* 0.05) or satisfaction with life (indirect effect = 0.021, 95% CI = [−0.018, 0.071], *P >* 0.05) did not mediate the effect of dispositional hope on left SMA GMV. Sex, age, family SES and total GMV were controlled for in these models. These findings suggested that there may be one possible pathway to influence subjective well-being, in which dispositional hope mediates the effect of GMV on subjective well-being.

## Discussion

To our knowledge, this is the first study to investigate the neurostructural correlates of hope and their associations with subjective well-being. Whole-brain regression analyses and prediction analyses showed that greater left SMA GMV was robustly linked to higher dispositional hope. Moreover, mediation analyses found that dispositional hope served as a mediator explaining the relationship between left SMA GMV and subjective well-being. Overall, this research revealed left SMA GMV as a neurostructural marker for dispositional hope and provided an underlying pathway for protecting subjective well-being in which dispositional hope mediates the link between GMV and subjective well-being.

First, we detected a positive relation between GMV of the left SMA and dispositional hope. These data fit well with the findings showing GMV abnormalities in the SMA in low hope-associated mental disorders, such as major depression disorder, anxiety disorder, posttraumatic stress disorder and obsessive-compulsive disorder (Radua and Mataix-Cols, 2009; Li *et al.,* 2014; Zhang *et al.,* 2016; Wang, Cheng *et al.,* 2018). As a well-known and essential cortical region for movement-related processing, the SMA is considered to be a critical structure linking cognition to action (Goldberg, 1985; Haggard, 2005; Nachev *et al.,* 2008). Particularly, the SMA has been demonstrated to be involved in a series of goal-directed behaviors, including goal expectation and selection, intention initiation and preparation, action planning and execution (Tanji and Shima, 1994; Matsumoto and Tanaka, 2004; Diedrichsen *et al.,* 2006; Makoshi *et al.,* 2011), which correspond to the pathway thinking of hope theory that underscores individuals’ abilities to produce different ways to solve problems and meet goals (Snyder, 2002). The SMA was also found to be crucial for motivating and selecting behavior and integrating motivation and cognitive control to make optimal decisions to achieve goals (Campos *et al.,* 2005; Egner, 2009; Kouneiher *et al.,* 2009). The motivational function of the SMA has self-evident relevance for the agency thinking of hope theory that highlights the role of motivation in pursuing a given goal (Snyder, 2002). Furthermore, there is evidence revealing that the functioning of the SMA is linked with several cognitive-emotional processes, including emotional regulation (Frank *et al.,* 2014), positive self-evaluation (Beer, 2007) and optimistic tendency (Wang, Zhao *et al.,* 2018), which are parallel psychological constructs highly associated with dispositional hope (Magaletta and Oliver, 1999; Snyder, 2002). Altogether, our finding regarding the association of left SMA with dispositional hope might reflect the role of SMA in goal-directed behavior, motivation and cognitive-emotional processing, which may make a significant contribution to the development of dispositional hope.

Critically, we revealed an indirect effect of dispositional hope on the association of left SMA GMV with subjective well-being. At the behavioral level, the relationship of dispositional hope with subjective well-being has been well established in previous studies (Ciarrochi *et al.,* 2007; Marques *et al.,* 2013; Ng *et al.,* 2014; Ciarrochi *et al.,* 2015; Heinitz *et al.,* 2018). This relationship was further confirmed in the current dataset even after adjusting for sex, age, family SES and total GMV. Thus, our finding may present further evidence that dispositional hope is a prominent personality resource for acquiring subjective well-being. At the neural level, we observed a positive association of left SMA GMV with subjective well-being. Although there are currently no reports of a relation between SMA structure and subjective well-being, there is some evidence indicating a role of SMA function in measures of subjective well-being (Volkow *et al.,* 2011; Luo *et al.,* 2014; Shi *et al.,* 2018). For example, using a regional homogeneity analysis method based on rsfMRI (Zang *et al.,* 2004), Luo *et al.* (2014) found decreased SMA spontaneous brain activity in happy participants compared with unhappy participants. One recent study further revealed that the SMA dynamic functional connectivity with other brain regions in the salience network can predict variance in subjective well-being (Shi *et al.,* 2018). Moreover, evidence from a transcranial magnetic stimulation study observed that transient disruption of the left SMA can disrupt participants’ facial happiness recognition, suggesting the role of SMA in facial happiness perception and experience (Rochas *et al.,* 2013). As mentioned above, the SMA is a core brain region for movement-related processing (Goldberg, 1985; Nachev *et al.,* 2008), particularly for physical activity or exercise (Voelcker-Rehage and Niemann, 2013), which has a prominent impact on subjective well-being (Fox, 1999; Pawlowski *et al.,* 2011; Wiese *et al.,* 2018). In view of the role of SMA in emotional processing and emotion-action interactions (Warren *et al.,* 2006; Etkin *et al.,* 2011; Blakemore and Vuilleumier, 2017), the involvement of the SMA may help individuals participate in more physical activity or exercise so that more positive emotion and higher levels of hope are developed, which may further enhance one’s subjective well-being. Overall, our findings indicated that dispositional hope may be a potential mechanism linking left SMA to subjective well-being.

Our research has several limitations. The first limitation was that the measures of dispositional hope and subjective well-being were based on self-report questionnaires and may be vulnerable to response bias. It is necessary for future studies to employ multiple techniques (e.g. qualitative interviewing or implicit testing) to lessen the impact of the response bias and improve measurement accuracy. Second, we found that only GMV in the left SMA was linked to dispositional hope and failed to find links between dispositional hope and GMV in other PFC regions that have been hypothesized to be associated with dispositional hope (Wang, Xu *et al.,* 2017). Because dispositional hope is a complex and abstract construct, it may involve multiple brain regions with relatively weak effects (i.e. the weak diffuse effects; Cremers *et al.,* 2017). In this study, our analysis method was a simply whole-brain correlation analysis based on a single brain structure measure and a relatively strict significance threshold, which may decrease the statistical power to detect these weak effects. Future studies are encouraged to use more advanced analysis strategies (e.g. hypothesis-driven ROI analyses, multivariate-based analyses and network analyses; Cremers *et al.,* 2017) with a less stringent significance threshold to improve statistical power to further examine the neuroanatomical basis of dispositional hope. Third, the correlational nature of the present study makes it impossible to draw causal inferences on the relations between GMV, dispositional hope and subjective well-being. Notably, the mediation tests performed in this study are of a statistical nature and do not imply causality. Future investigations should utilize more sophisticated methods (e.g. longitudinal or intervention designs) to establish the causal direction of the relations between these constructs. Additionally, the follow-up analyses in this study were based on a ROI (i.e. SMA) that was specifically related to dispositional hope and selected from the initial whole-brain regression analysis, which may lead to the problem with circular analyses and bias the results. For example, the *r*-value for the prediction analysis based on the same participants was likely to be higher than it would be by chance. Similarly, the SMA-hope relation in the meditation models may be inflated, which may bias results away from the other potential relations. Thus, future studies are warranted to use independent data and analyses to validate our findings.

In conclusion, we provide initial evidence for a neurostructural marker underlying dispositional hope, as revealed by its link to GMV in the left SMA. Moreover, we present new evidence indicating that dispositional hope mediates the link between left SMA volume and subjective well-being. These findings jointly suggest the key role of dispositional hope and GMV in the development of subjective well-being, and they also introduce new study directions for exploring how brain features influence subjective well-being through individual psychological attributes. Additionally, our findings may have implications for potential neural (e.g. Salehinejad *et al.,* 2017) and behavioral (e.g. Feldman and Dreher, 2012) interventions to cultivate adolescents’ hope and enhance their subjective well-being. Finally, our research may advance the development of psychoradiology (https://radiopaedia.org/articles/psychoradiology), a frontier of radiology aiming at uncovering abnormal functional and structural brain changes in psychiatric disorders and also guiding clinical diagnosis and treatment planning decisions in these disorders (Lui *et al.*, 2016; Huang *et al.*, 2019; Gong, 2020).

## Funding

This study was funded by the National Natural Science Foundation of China (Grant Nos. 31800963, 81621003 and 81820108018), the Program for Changjiang Scholars and Innovative Research Team in University (PCSIRT, Grant No. IRT16R52) of China, the China Postdoctoral Science Foundation (Grant No. 2019 M653421), and the Postdoctoral Interdisciplinary Research Project of Sichuan University. Dr Gong would also like to acknowledge the support from his Changjiang Scholar Professorship Award (Award No. T2014190) of China and the American CMB Distinguished Professorship Award (Award No. F510000/G16916411) administered by the Institute of International Education, USA.

## Conflict of interest

None declared .

## Supplementary Material

Supplemental_materials_nsaa046
